# N^6^-(2-Hydroxyethyl)-Adenosine Exhibits Insecticidal Activity against *Plutella xylostella* via Adenosine Receptors

**DOI:** 10.1371/journal.pone.0162859

**Published:** 2016-09-26

**Authors:** Ming Fang, Yiqiu Chai, Guanjv Chen, Huidong Wang, Bo Huang

**Affiliations:** 1 Key Laboratory of Entomogenous Fungi Resources Research and Development of Wenzhou, Zhejiang Institute of Subtropical Crops, Zhejiang Academy of Agricultural Sciences, Wenzhou, Zhejiang 325005, China; 2 Research Center for Entomogenous Fungi, Anhui Agricultural University, Hefei, Anhui 230036, China; 3 College of Plant Protection, Nanjing Agricultural University, Nanjing 210095, China; Natural Resources Canada, CANADA

## Abstract

The diamondback moth, *Plutella xylostella*, is one of the most important pests of cruciferous crops. We have earlier shown that N^6^-(2-hydroxyethyl)-adenosine (HEA) exhibits insecticidal activity against *P*. *xylostella*. In the present study we investigated the possible mechanism of insecticidal action of HEA on *P*. *xylostella*. HEA is a derivative of adenosine, therefore, we speculated whether it acts via *P*. *xylostella* adenosine receptor (*PxAdoR*). We used RNAi approach to silence *PxAdoR* gene and used antagonist of denosine receptor (AdoR) to study the insecticidal effect of HEA. We cloned the whole sequence of *PxAdoR* gene. A BLAST search using NCBI protein database showed a 61% identity with the *Drosophila* adenosine receptor (DmAdoR) and a 32–35% identity with human AdoR. Though the amino acids sequence of *PxAdoR* was different compared to other adenosine receptors, most of the amino acids that are known to be important for adenosine receptor ligand binding and signaling were present. However, only 30% binding sites key residues was similar between PxAdoR and A1R. HEA, at a dose of 1 mg/mL, was found to be lethal to the second-instar larvae of *P*. *xylostella*, and a significant reduction of mortality and growth inhibition ratio were obtained when HEA was administered to the larvae along with *PxAdoR*-dsRNA or antagonist of AdoR (SCH58261) for 36, 48, or 60 h. Especially at 48 h, the rate of growth inhibition of the *PxAdoR* knockdown group was 3.5-fold less than that of the HEA group, and the corrected mortality of SCH58261 group was reduced almost 2-fold compared with the HEA group. Our findings show that HEA may exert its insecticidal activity against *P*. *xylostella* larvae via acting on PxAdoR.

## Introduction

Adenosine is an endogenous nucleoside that modulates numerous physiological processes, including oxygen and metabolic balance in tissues. Intracellular adenosine is an important intermediate in the biosynthetic pathway that generates adenosine triphosphate (ATP). However, the nucleoside can also be released from cells or formed extracellularly from ATP, adenosine diphosphate (ADP), and adenosine monophosphate (AMP). The extracellular adenosine acts as a stress hormone, causing neuromodulation, vasoconstriction [1–2]. Adenosine regulates cellular functions by binding to G protein coupled receptors (GPCRs). Four mammalian subtypes of the AdoR, namely, A1, A2A, A2B, and A3, have been identified, and their genes have been cloned. The subtypes have been shown to modulate intracellular levels of adenosine 3’,5’-cyclic monophosphate (cAMP) in different ways [3]. In certain cells, the adenosine receptor (AR) is also coupled to the calcium-mobilizing G protein subunit, G_αq_ [4]. The adenosine derivative N^6^-(2-hydroxyethyl)-adenosine (HEA) was first isolated from the *Cordyceps pruinosa* and was identified as a calcium antagonist that inhibits muscle contraction [5].

The diamondback moth, *Plutella xylostella*, is one of the most important pests of cruciferous crops. The use of chemical insecticides is the preferred method for its control, however, it leads to the development of insecticide-resistance among pests and poses concern over possible environmental and human health hazards. Therefore, a search for new insect-control agents is highly warranted. Entomopathogenic fungi may be an alternative source of insect-control agents because these fungi constitute a rich source of bioactive chemicals [6].

Our earlier study has shown that the *Paecilomyces cicadae*, an entomopathogenic fungi, exerts an insecticidal effect on diamondback moth, *Plutella xylostella* [7]. The insecticidal active compound was isolated from the fruiting body of *P*. *cicadae* which is often used as traditional Chinese medicine, and was identified to be HEA [8]. Most of the published articles of HEA focus on humans, no published studies reporting insecticidal activity of HEA apart from ours [9]. It has been shown that overexpression of *DmAdoR* (increasing adenosine) *in vivo* causes lethality or severe developmental anomalies on *Drosophila melanogaster* [10–12]. We are interested in whether HEA, an adenosine derivative, has insecticidal activity to *P*. *xylostella* via PxAdoR. Therefore, we examined the *PxAdoR* gene expression, growth and mortality of *P*. *xylostella* by down regulating *PxAdoR* gene function using RNAi technology and AR inhibitor.

## Materials and Methods

### Insect strains

Susceptible strains of *P*. *xylostella* 2 instar larvae were provided by Nanjing Agricultural University. The moths were reared on Chinese cabbage in the laboratory in the Institute of Plant Protection, Zhejiang Institute of Subtropical Crops, at 25 ± 1°C, 16 h/8 h light/dark photoperiod and 65 ± 5% relative humidity.

### RNA extraction and cDNA synthesis

Total RNA was extracted from the whole bodies of *P*. *xylostella* second instar larvae using Trizol reagent (Invitrogen/Life Technologies, Paisley, UK) as per manufacturer's instructions and then quantitated by calculating the absorbance ratio and by agarose gel electrophoresis. First strand cDNA was synthesized from 2 μg of RNA with the RevertAid First Strand cDNA Synthesis Kit K1622 (Thermo Scientific, US) by following the manufacturer's protocol.

### Cloning of Full-length *PxAdoR* cDNA

The mRNA was reverse transcribed to cDNA with the common primer named Oligo (dT), and the Gene Specific Primers (GSPs) were generated for the amplifications. A second set of primers that extend from the unknown end of the message back to the known region of the 3’end was provided by the poly (A) tail, while an appended homopolymer tail was used for the 5’end [13]. Degenerate primers PxTA-F and PxTA-R (Table 1) were designed for the amplification of a partial *PxAdoR* cDNA. A set of specific primers was synthesized based on the sequence of putative insert for 5'- and 3'-rapid amplification of cDNA ends (RACE). rCai-R1 and rCai-R2 were used for 5'-RACE, and rCai-F1 and rCai-F2 (Table 1) were used for 3'-RACE. The Reverse Transcriptase-Ploymerase Chain Reaction (RT-PCR) and PCR were uesd to amplify the ends of transcripts. RACE was performed by using the 5'-Full RACE Kit and 3'-Full RACE Core Set Version 2.0 (TakaRa, Dalian, China) according to the manufacturer's protocol. RACE products were gel purified and sequenced by Sangon Biotech Company (Shanghai, China).

**Table 1:** List of primers used in the study

Primer name	Primer sequence 5’-3’
PxTA-F	GAGCGAGGTGATGACGGTGGA
PxTA-R	GACGATGATGGCGAGGTTCTGC
rCai-F1	GCAGATGAGCGAGGTGATGACGGTG
rCai-F2	CCTCGCCATCATCGTCTTCTTCTTCATCA
rCai-R1	GGCGAGGTTCTGCGTGGCCTTGAC
rCai-R2	CACCGTCATCACCTCGCTCATCTGCTT
Pxds-F	CCCGAAGGACGAGACAA
Pxds-R	CAGCGAAACAACATTACCAC
Xiaocaiye-18F	GACTCAACACGGGAAATCTCACCA
Xiaocaiye-18R	CCAGACAAATCGCTCCACCAACTA

### Bioinformatic analysis

Comparative and bioinformatic analyses of nucleotide sequences and deduced amino acids sequences were carried out at http://www.ncbi.nlm.nih.gov and http://cn.expasy.org. The nucleotide sequence, deduced amino acids sequence, and open reading frame (ORF) were analyzed, and sequence comparison was conducted through database searches using BLAST programs (http://www.ncbi.nlm.nih.gov/BLAST/). The phylogenetic analysis of *PxAdoR* from other species was done by Clusta X program version 1.83 using default parameters [14] and manual adjustment where necessary. A phylogenetic tree was constructed using MEGA (molecular evolutionary genetics analysis) program, version 4.0 [15] from Clustal W1.6 alignment.

### *PxAdoR* Gene Silencing

A double-stranded RNA (348 bp) corresponding to a portion of the *PxAdoR* was synthesized by using a method that eliminates the cloning step [16]. Here, templates for *in vitro* transcription were produced by adding T7 promoter sequences to each 5’ end of cDNA template prepared by SMART-RACE cDNA Amplification Kit (Clontech) through polymerase chain reaction (PCR). The primers Pxds-F and Pxds-R (Table 1) used to generate a cDNA with T7 promoter sequences, were designed based on the *PxAdoR* sequence at positions 1314–1661. The PCR products were examined on agarose gel prior to in *vitro* transcription to verify that the products show a single band and the expected sizes. All dsRNA preparations were quantified spectrophotometrically and stored at −20°C until use.

Second instar larvae of *P*. *xylostella* were starved for 24 h and fed for 48 h with cabbage dishes which were cut into 10 cm diameter to fit the size of a petri dish [17], and drenched in 500 ng/mL [17]of Pxds or green fluorescent protein (*GFP*) -dsRNA 5 mins, and aired. Being a nontoxic reporter gene, GFP-dsRNA (dsGFP) was used as a control.

### Analysis of silencing specificity

To assess the off-target effects of the silencing method used in this study, we checked the expression levels [17] of *PxAdoR* along with dsGFP control by Quantitative RT-PCR 48 h after RNAi. qRT-PCR assays were performed in a 25 μL reaction volume by using SYBR premix Ex-Taq Perfect Real-time Kit (TakaRa, Dalian, China), with 10 pmol of primers and 20 ng total RNA-equivalent of each cDNA generated by reverse transcription in a Thermal Cycler Dice Real-Time System ABI7500 (Applied Biosystems, USA) with Primers for *PxAdoR* target sequences, as well as for endogenous 18S ribosomal RNA control, were designed by prime 5.0 (Table 1). Serial (10×) dilutions of cDNA samples were prepared to generate the relative standard curves for each of the genes tested for each qRT-PCR run. The thermal cycling conditions are as follows: 95°C for 10 s, followed by 40 cycles of 95°C for 5 s and 60°C for 30 s. A dissociation step at 95°C for 15 s and 60°C for 30 s was added as a final step for melt curve analysis. The cycle threshold (*C*_t_) value was used to calculate the transcript quantity of target genes on the basis of the standard curve. All calculations were performed by the software that accompanies the employed qRT-PCR machine. Each qPCR measurement was carried out independently for a minimum of three times, and the mean value was used for quantification. The 2^−△△*C*t^ method was used to analyze the relative changes in gene expression. The expression of the 18S gene was used as a control and the larvae fed the dsGFP were used as the negative control.

### Statistical analysis

The results are presented as means ± standard deviation (SD). Statistical analysis was performed using analysis of variance (ANOVA) followed by LSD test or Student’s t-test using SPSS ver. 20.0 software. A significant difference was defined as P < 0.05 and very significant difference was defined as P < 0.01.

### Effect of RNAi and receptor inhibitor on the insecticidal activity of HEA

Cabbages were cut into 10 cm diameter to fit the size of a petri dish, drenched in 1 mg/mL [9] HEA for 30 s, and aired. The second instar larvae were first allowed to feed on dsRNA for 48 hours and then ten of these larvae were transferred to cabbage treated with 1mg/ml HEA in a petri dish.

In the same method, the AR inhibitor SCH58261 was used as a positive control and the untreated larvae were used as a negative control. Each group consisted of five replicates. The mortalities and rate of growth inhibition were recorded after 24, 36, 48, and 60 h. Corrected mortality (%) = (mortality of treatment−mortality of control)/(1−mortality of control)×100. Rate of growth inhibition (%) = [(weight of control−weight of treatment)/ weight of control]×100.

## Results

### Cloning and Characterization of the *PxAdoR* Gene from *P*. *xylostella*

Transcriptome sequencing as part of our earlier study provided a 297 bp fragment. BLAST analysis showed that the nucleotide sequence of this fragment shared about 82% and 77% identities with *Papilio machaon* AR A2b-like and *Nasonia vitripennis* AR A2b.

**Fig 1:**
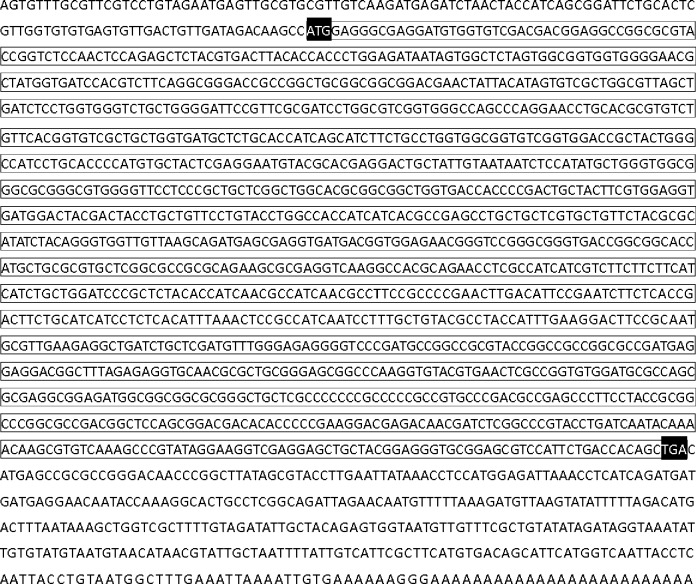
cDNA sequence of *Plutella xylostella* Adenosine Receptor. The line box area indicates the open reading frame (ORF), which encodes protein of 440 amino acids. The start codon (ATG) and the stop codon (TGA) are highlighted in black. The sequence was deposited in the GenBank (Assession No.KR258794).

Based on the sequence information, specific primers were designed for 5'-and 3'- RACE of the related gene. 5'-RACE generated a 784 bp fragment, and 3'-RACE produced a 960 bp fragment. Splicing the sequences generated a 1828 bp fragment, which was identified as the full-length *PxAdoR* gene (GenBank accession NO.KR258794). The ORF of *PxAdoR* cDNA encoded a protein of 440 amino acids residues with a calculated molecular mass of 64.74243 kDa and an isoelectric point of 11.495 (Fig 1).

### Alignment of *P*. *xylostella* AdoR with other known AdoRs

After the sequences of the *PxAdoR* gene were determined, the evolutionary position of the gene among the various *AdoR* genes was investigated. The sequence of *PxAdoR* was submitted to NCBI for BLAST searches, and results showed that *PxAdoR* presents some homology with insect *AdoR* genes from other species, with 78% identity with *Papilio machaon AdoR*, 77% and 78% identity with *Amyelois transitella* and *Papilio xuthus AdoR*, and 61% identity with *Drosophila melanogaster AdoR*. The gene exhibited low similarity with human AdoR, with 33% identity with human A1R, 35% identity with A2aR, 32% identity with A2bR, and 34% identity with A3R (Table 2).

**Table 2:** The similarity of protein sequences between PxAdoR and other insect adenosine receptors.

Description	Query cover	Identities
PREDICTED: adenosine receptor A2b-like [*Papilio xuthus*]	100%	78%
PREDICTED: adenosine receptor A2b-like [*Papilio machaon*]	100%	78%
PREDICTED: adenosine receptor A3 [*Amyelois transitella*]	99%	77%
PREDICTED: adenosine receptor A2b [*Bombyx mori*]	99%	73%
Adenosine receptor A2a [*Papilio xuthus*]	68%	74%
Adenosine receptor A2a [*Papilio machaon*]	68%	74%
GH18390 [*Drosophila grimshawi*]	80%	60%
hypothetical protein KGM_02847 [*Danaus plexippus*]	68%	72%
hypothetical protein FF38_04892 [*Lucilia cuprina*]	79%	62%
PREDICTED: uncharacterized protein LOC106087647 [*Stomoxys calcitrans*]	78%	62%
PREDICTED: uncharacterized protein LOC105233440 [*Bactrocera dorsalis*]	82%	58%
adenosine receptor, isoform B [*Drosophila melanogaster*]	82%	61%
PREDICTED: LOW QUALITY PROTEIN: adenosine receptor A2b [*Plutella xylostella*]	47%	99%
PREDICTED: adenosine receptor A2b [*Apis mellifera*]	68%	68%
PREDICTED: adenosine receptor A2b-like [*Megachile rotundata*]	70%	68%
PREDICTED: adenosine receptor A2b [*Nasonia vitripennis*]	68%	69%
hypothetical protein L798_01328 [*Zootermopsis nevadensis*]	83%	60%
AAEL002894-PA [*Aedes aegypti*])	67%	65%
PREDICTED: adenosine receptor A2b-like isoform X1 [*Wasmannia auropunctata*]	66%	64%
PREDICTED: adenosine receptor A2b-like isoform X1 [*Vollenhovia emeryi*]	66%	65%
Adenosine receptor A2a [*Operophtera brumata*]	60%	72%
PREDICTED: adenosine receptor A2a [*Tribolium castaneum*]	83%	57%
adenosine receptor A2a [*Homo sapiens*]	65%	35%
adenosine receptor A2b [*Homo sapiens*]	65%	32%
adenosine receptor A3 isoform A [*Homo sapiens*]	65%	34%
adenosine receptor A1 [*Homo sapiens*]	68%	33%

**Fig 2:**
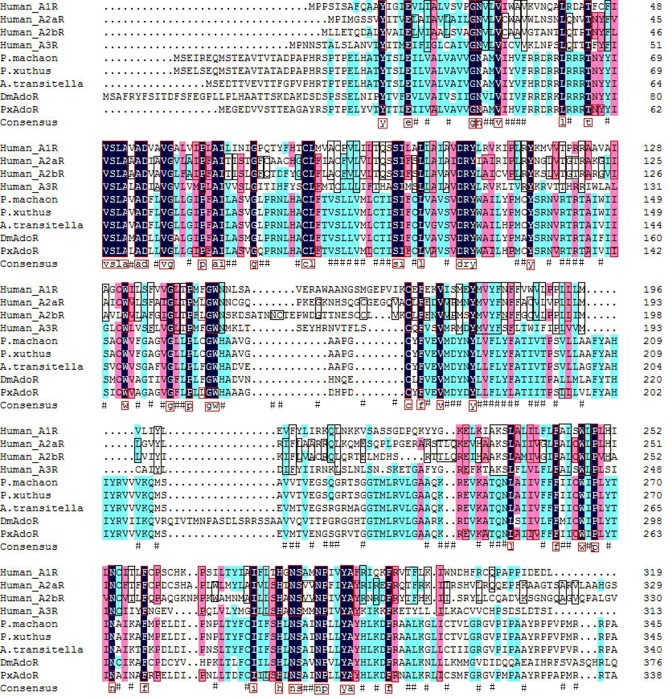
Alignment of the human A2b adenosine receptor and N-terminal part of AdoR amino acids sequences of other insects and *Plutella xylostella*. Different color shows different homology levels. Black means 100% homology level, pink shows 75% homology level, blue stands for 50% homology level, and yellow marks 33% homology level. The PxAdoR and human adenosine receptors share the same key residues marked by red solid border and the mismatched key residues between PxAdoR and human adenosine receptors are marked by black solid border. “#” sign indicates the special residues of PxAdoR compared with human adenosine receptors.

The predicted ORF of the *PxAdoR* gene encodes a protein of 440 amino acids. Using the method of Clustal W, a comparison of amino acids sequence showed that the N-terminal sequence of PxAdoR had a low homology with human A2b adenosine receptor, but was more similar with *Drosophila* AdoR and other insects AdoRs. The human adenosine receptor have 193 binding sites key residues [18], only 71 binding sites key residues of A1R, 84 of A2aR, 85 of A2bR, and 64 of A3R are identical compared to that of PxAdoR (Fig 2).

The PxAdoR belongs to the G protein coupled receptors, has seven transmembrane helices [19] and contains the most important amino acids for adenosine receptor ligand binding [20–21].

### *PxAdoR* silencing and specificity

**Fig 3:**
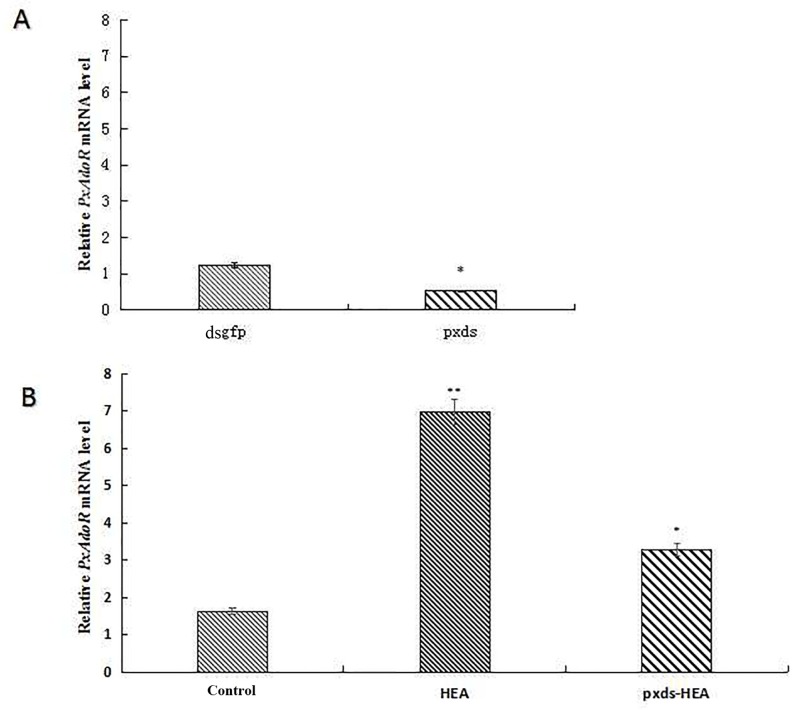
Relative *PxAdoR* mRNA level. **(A) Effect of *PxAdoR* silencing by 500 ng/mL dsRNA feeding. (B) Effect of HEA feeding after silencing *PxAdoR*.** Analyzed sample were cDNAs that were reverse-transcribed from pooled RNA samples of 10 larvae. Bars represent the means ± standard deviations of technical replicates. *P < 0.05; **P < 0.001 (A) compared with the dsgfp group. (B) compared with the control.

Quantitative RT-PCR analyzes the RNAi experiments showed that *PxAdoR* gene expression in the second instar larvae after RNAi was reduced by 2.36-fold compared to dsGFP-fed control (Table 3). The findings show that dsRNA feeding resulted in a significantly reduced the expression of *PxAdoR* gene (Fig 3A).

**Table 3:** The effect on relative *PxAdoR* mRNA level by qRT-PCR.

Sample Name	△Ct (n = 3)	Relative PxAdoR mRNA level (2^-△△Ct^) (n = 3)
dsGFP1	22.153±0.144	1.239±0.664
dsGFP2	22.612±0.357	
dsGFP3	21.161±0.087	
Pxds1	22.818±0.133	0.525±0.111
Pxds2	23.057±0.113	
Pxds3	23.443±0.267	
Control1	17.893±0.069	1.611±0.543
Control2	16.868±0.132	
Control3	17.047±0.422	
HEA1	14.996±0.018	6.850±0.584
HEA2	15.125±0.064	
HEA3	15.241±0.556	
Pxds-HEA1	16.207±0.096	3.652±1.099
Pxds-HEA2	15.600±0.412	
Pxds-HEA3	16.390±0.026	

After treatment with 1 mg/mL HEA, the relative *PxAdoR* mRNA level showed a highly significant, almost 4.3-fold (*P* < 0.001) increase compared to the control (Table 3). The relative *PxAdoR* mRNA level of Pxds-HEA group exhibited a significantly reduced expression in comparison with the HEA group but was higher than the control. This data shows that HEA affects *PxAdoR* gene expression and suggests that it could induce insecticidal activity via *P*. *xylostella* AdoRs (Fig 3B).

### Effect of RNAi and receptor inhibitor on the insecticidal activity of HEA on *P*. *xylostella* larvae

**Fig 4:**
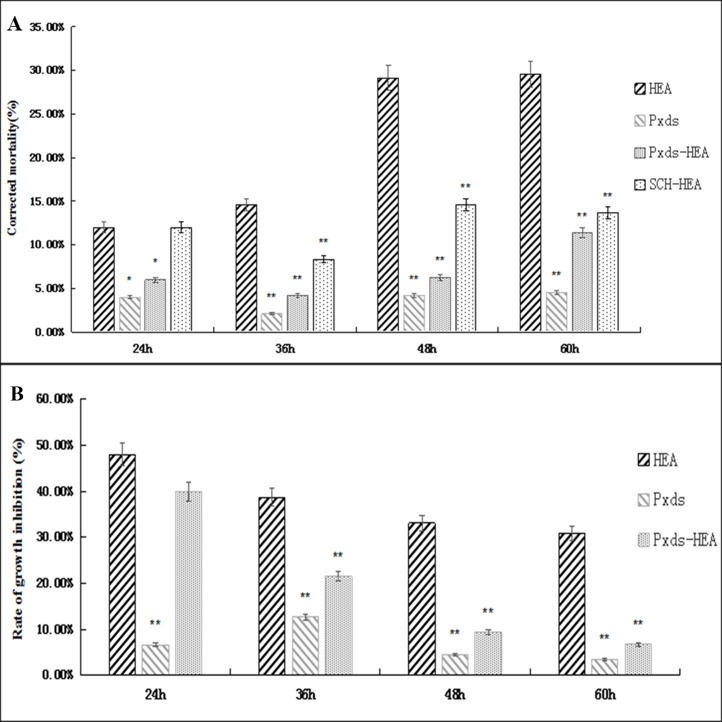
Impact of RNAi and Receptor Inhibitor on the insecticidal effect of HEA. Larvae were fed with dsRNA or receptor inhibitor (SCH58261) with or without HEA for a period of 24 h to 60 h. *P < 0.05; **P < 0.001 compared with the HEA group. (A) Effect of *PxAdoR* silencing and SCH58261 on the mortality induced by HEA. Corrected mortality (%) = (mortality of treatment−mortality of control)/(1−mortality of control)×100. (B) Effect of *PxAdoR* silencing on the rate of larvae growth. Rate of growth inhibition (%) = [(weight of control−weight of treatment)/ weight of control]×100.

The mortality and rate of growth inhibition of the group fed with Pxds were the lowest compared to control group and the corrected mortality rates were found to be below 5% at 24, 36, 48, and 60 h time points. This data shows that Pxds feeding affected the insect body minimally. The corrected mortality of the Pxds–HEA group showed a significant reduction at all time points (24 h: from 12% to 6%; *P* < 0.05; 36 h: 14% to 4%; P < 0.001; 48 h: 29% to 6%; P < 0.001; 60 h: 29% to 11%; P < 0.001) compared with the HEA group at each time point. These results show that silencing *PxAdoR* gene can reduce the lethal effect of HEA on *P*. *xylostella* larvae. The corrected mortality of the SCH-HEA group at 24 h did not show a significant difference compared to the HEA group, however, a significant reduction in mortality was observed from 36 h to 60 h time points (*P* < 0.001). In particular, at 48 and 60 h, almost 2-fold reduction in mortality was observed (Fig 4A).

The rate of growth inhibition of the Pxds–HEA group at 24 h was not significantly (*P* > 0.05) different compared to the HEA group. However, a statistically significant (*P* < 0.001) reduction was observed at 36 h to 60 h (36 h: 38% to 21%; 48 h: 33% to 9%; 60 h: 30% to 6%) (Fig 4B). This shows that the insecticidal activity of HEA on *P*. *xylostella* larvae is via PxAdoR.

## Discussion

In this study we investigated the possible underlying mechanism of insecticidal activity of HEA on *P*. *xylostella*. qRT-PCR and RACE was used to clone the gene of *P*. *xylostella* adenosine receptor (*PxAdoR*). According to the Franchetti’s study that A1R had 10 key binding sites residues (M180, L88, T91, H278, T277, W247, N254, L258, F185, V181) interacting with N6 derivatives CPA which was the agonist of A1R [22]. Only 3 residues of PxAdoR (H278, W247, N254) were same as that of A1R, also the similarity was 30%. We speculated that HEA which was the N6 derivatives adenosine has the same binding sites as CPA. Activation of A1R of human and mammals has effect of sedative, hypnotic, anticonvulsant [23], antiinflammatory [24]. HEA has the same effect for human through activating A1R.[25,26]. Thus, HEA could be a possible environmentally safe biological pesticide that is nontoxic to the human body.

Since *PxAdoR* exhibited considerable homology with AdoRs of other insects, this provides a possibility of selecting adenosine-like insecticides through other insect AdoRs e.g. adenosine receptor agonists 5’-*N*-Ethylcarboxamidoadenosine (NECA), N^6^-Cyclopentyladenosine (CPA), 2-Chloro-N6-cyclopentyladenosine (CCPA), and non-purine adenosine receptor agonist Paeoniflorin. This can further broaden the adenosine-AdoR insecticide spectrum.

Based on the sequence of the receptor gene, we designed specific primers for a double-stranded RNA synthesis. After dsRNA feeding to reduce the expression of *PxAdoR*, *P*. *xylostella* were fed with HEA and receptor antagonist SCH58261. The corrected mortality and the rate of growth inhibition were analyzed to find whether HEA acts through *PxAdoR*. Our data showed that Pxds–HEA group, with larvae that were fed with dsRNA and HEA, had a significantly lower mortality and rate of growth inhibition compared to the group treated with only HEA, thus showing that HEA acts through PxAdoR.

HEA was first isolated from the entomopathogenic fungi *C*. *pruinosa* and it was identified to be a calcium antagonist. That instance marked the first time that a synthetically known compound has been isolated biologically [5]. As part of our previous study we showed that *P*. *cicadae* exerts an insecticidal effect on diamondback moth, *P*. *xylostella* and isolated the insecticidal active compound, HEA, from the fruiting body of *P*. *cicadae*, which is used in ancient traditional Chinese medicine [9].

Based on our previous observations, we assessed the possibility of using HEA as a pesticide against the diamondback moth, *P*. *xylostella*. Being an adenosine derivative, HEA will be contacted with AdoRs. In a previous study, Tomas Dolezal *et al*. have shown that varying adenosine levels in *Drosophila in vivo* results in metamorphic changes such as developmental delay or fat body disintegration [27]. Another study by Monika Zuberova *et al*. showed that increasing extracellular adenosine levels in *Drosophila* affects the energy stores leading to wasting and death [10].

To address the question whether HEA, an adenosine derivative, induces insecticidal effects through its interaction with AdoRs, we used RNAi silencing of *PxAdoR* gene by feeding *P*. *xylostella* with specific dsRNA [28,17]. Our findings show that feeding dsRNA reduces expression of *PxAdoR* gene. We found that RNAi exerts evident effect from 24 h to 60 h after feeding dsRNA. Silencing of PxAdoR gene through RNAi could inhibit the lethal effect of HEA and the growth of *P*. *xylostella* larvae at 36–60 h. Further, treatment with an antagonist of A2A receptor could also weaken the effect of HEA on *P*. *xylostella*. These findings show that the HEA acts against *P*. *xylostella* via adenosine receptors.

Magazanik *et al*. studied the effects of adenosine and ATP on blowfly larvae, *Calliphora vicina*, and found that presynaptic AdoRs could regulate transmitter release at insect motor nerve terminals [29]. This finding showed that adenosine modulates neuronal activity via AdoRs. To determine if adenosine modulates neuronal activity in invertebrate neurons, Malik *et al*. conducted whole-cell recordings and found that adenosine can depress neuronal activity via AdoRs [30]. It could also be due to elevated Ca^2+^ due to the inactivation of N-methyl-D-aspartate receptors [31], which are Ca^2+^ permeable glutamate-gated ion channels that are regulated by [Ca^2+^] and are necessary for fast excitatory neurotransmission in the central nervous system [32]. It is known that adenosine activates the AR through the second messenger cAMP for Ca^2+^ control. However, there are no similar studies on PxAdoR. Therefore, as part of our future studies, we intend to investigate how the HEA effect on second messenger cAMP and Ca^2+^ by activation of AR of *P*. *xylostella*. This approach is expected to provide an in-depth understanding of the insecticidal mechanism of HEA.

In conclusion, our findings show that HEA acts on *P*. *xylostella* and induces insecticidal activity through PxAdoR, *P*. *xylostella* adenosine receptors. Since PxAdoR has a very low homology with human AdoRs, HEA can potentially be used as an environmentally safe pesticide against the diamondback moth, *P*. *xylostella*, further more, may screening pesticide with PxAdoR.
